# Prognostic importance of modified geriatric nutritional risk index in oral cavity squamous cell carcinoma

**DOI:** 10.1038/s41598-024-63671-y

**Published:** 2024-06-05

**Authors:** Yao-Te Tsai, Ming-Hsien Tsai, Geng-He Chang, Ming-Shao Tsai, Ethan I. Huang, Chang-Hsien Lu, Cheng-Ming Hsu, Chia-Hsuan Lai, Chun-Ta Liao, Chung-Jan Kang, Yi-Chan Lee, Yuan-Hsiung Tsai, Ku-Hao Fang

**Affiliations:** 1 Department of Otorhinolaryngology-Head and Neck Surgery, Chang Gung Memorial Hospital, Chiayi, Taiwan; 2 College of Medicine, Chang Gung University, Taoyuan, Taiwan; 3 Department of Otorhinolaryngology-Head and Neck Surgery, Chang Gung Memorial Hospital, Kaohsiung, Taiwan; 4 Department of Hematology and Oncology, Chang Gung Memorial Hospital, Chiayi, Taiwan; 5 Department of Radiation Oncology, Chang Gung Memorial Hospital, Chiayi, Taiwan; 6 Department of Otorhinolaryngology-Head and Neck Surgery, Chang Gung Memorial Hospital, Taoyuan, Taiwan; 7 Department of Otorhinolaryngology-Head and Neck Surgery, Chang Gung Memorial Hospital, Keelung, Taiwan; 8 Department of Diagnostic Radiology, Chang Gung Memorial Hospital, Chiayi, Taiwan; 9 Chang Gung Memorial Hospital, No.6, W. Sec., Jiapu Rd., Puzi City, Chiayi County 613 Taiwan

**Keywords:** Biomarkers, Oncology

## Abstract

We probed the associations of preoperative modified geriatric nutritional risk index (mGNRI) values with prognosis in patients receiving surgery for oral cavity squamous cell carcinoma (OCSCC). This retrospective study analyzed the clinical data of 333 patients with OCSCC and undergoing surgery between 2008 and 2017. The preoperative mGNRI was calculated using the following formula: (14.89/C-reactive protein level) + 41.7 × (actual body weight/ideal body weight). We executed receiver operating characteristic curve analyses to derive the optimal mGNRI cutoff and employed Kaplan–Meier survival curves and Cox proportional hazard model to probe the associations of the mGNRI with overall survival (OS) and disease-free survival (DFS). The optimal mGNRI cutoff was derived to be 73.3. We noted the 5-year OS and DFS rates to be significantly higher in the high-mGNRI group than in the low-mGNRI group (both *p* < 0.001). A preoperative mGNRI below 73.3 was independently associated with unfavorable DFS and OS. A mGNRI-based nomogram was constructed to provide accurate OS predictions (concordance index, 0.781). Hence, preoperative mGNRI is a valuable and cost-effective prognostic biomarker in patients with OCSCC. Our nomogram facilitates the practical use of mGNRI and offers individualized predictions of OS.

## Introduction

Oral cavity cancer is the most prevalent type of head and neck cancer, and its global incidence is increasing^1^. In particular, 377,713 and 177,757 new diagnoses of and deaths due to oral cavity cancer were recorded worldwide in 2020^1^. From a histological viewpoint, over 90% of oral cavity cancer cases are accounted for by oral cavity squamous cell carcinoma (OCSCC)^2^. At present, curative surgery is the cornerstone of treatment for OCSCC, whether administered singly or in conjunction with adjuvant therapy^3^. In situations where surgery is not feasible, definitive chemoradiotherapy (CRT) is considered^4^. In addition to tumor–node–metastasis (TNM) stage, the survival outcomes of OCSCC can be influenced by a variety of tumor characteristics, such as extranodal extension (ENE), poor differentiation (P-D), lymphovascular invasion (LVI), perineural invasion (PNI), depth of invasion (DOI), as well as patient-level factors, including malnutrition and systemic inflammation^5–9^. Nevertheless, data on most of these clinicopathological factors can be obtained only after surgery. Thus, easy-to-measure and novel preoperative biomarkers must be identified for the early formulation of optimized treatment strategies.

The geriatric nutritional risk index (GNRI) was initially introduced to determine the prognosis of hospitalized older adult patients on the basis of their height, body weight, and serum albumin level^7^. Several studies, involving younger and older adult patients, have indicated that the GNRI is a straightforward nutrition-related indicator for determining prognoses in various types of cancer, including late-stage OCSCC^10–13^. However, the serum albumin level can fluctuate easily and is influenced by various factors, such as chronic liver disease, corticosteroid use, and body fluid imbalance^14^. Studies also suggest that depending solely on body mass index (BMI) may not be sufficient for identifying malnutrition in OCSCC patients, as it fails to consider variations in body composition^15^. A growing body of evidence indicates a close association between inflammatory responses within tumor microenvironments and key aspects of cancer, such as its development, progression, and distant metastasis^16^. C-reactive protein (CRP) is a widely recognized acute-phase protein that serves as a marker of systemic inflammatory response and is linked to the prognosis of patients with OCSCC^17,18^. In addition, studies have indicated that measuring preoperative CRP may assist in predicting the pathological aggressiveness of OCSCC and the complications of flap reconstruction^19,20^. Recently, Kouzu et al. introduced a modified version of the GNRI (mGNRI), in which they replaced albumin with inverse CRP^21^. They demonstrated that the mGNRI offered improved prognostic discrimination relative to the GNRI in patients with esophageal cancer undergoing esophagectomy. The prognostic utility of the mGNRI has also been demonstrated in various forms of cancer^22–24^. Nevertheless, the prognostic utility of the mGNRI in OCSCC remains uncertain. Hence, in this study, we explored the potential utility of the mGNRI as a prognostic indicator for OCSCC. Because the mGNRI is indicative of a patient’s nutritional status and systemic inflammatory responses, we hypothesized that preoperative mGNRI values would exhibit a strong association with survival outcomes in OCSCC and can thus be used for screening and treatment planning. We also incorporated a variety of clinicopathological variables and the mGNRI to create a nomogram and evaluated the nomogram’s performance in predicting individual survival outcomes.

## Methods

### Study population

Initially, the medical data of 357 consecutive patients with primary OCSCC in our hospital for the period between December 2017 and January 2008 were retrospectively retrieved. We included (1) patients who received a diagnosis of OCSCC based on histopathological evidence and (2) patients who underwent ablative surgery as primary treatment. Regarding patient exclusion, we removed those having a history of malignancy (n = 5), having synchronous cancer at the diagnosis of OCSCC (n = 4), with suspected acute inflammation or infection at the time of laboratory examinations (n = 3), having a history of preoperative neoadjuvant treatment (n = 5), or with missing follow-up data (missing at random, n = 7). Accordingly, we included in our study analysis a total of 333 patients. This retrospective study was executed in accordance with the principles outlined in the Declaration of Helsinki, and before executing our study, we received approval from the institutional review board of Chang Gung Memorial Hospital (number: 202301163B0). Prior to being included in our study analysis, each of the patients provided their written informed consent.

### Data collection

The patients’ electronic medical records were collected and reviewed by medical staff. Thorough evaluations were conducted to obtain clinical data on the following clinicopathological characteristics: age at diagnosis of OCSCC; PNI status; sex; ENE status; tumor subsites and grading; pathological cancer stage (recorded on the basis of the American Joint Committee on Cancer [AJCC] staging manual, 8th edition, 2018); DOI; nearest resection margin; underlying comorbidities (classified on the basis of the Charlson comorbidity index [CCI])^25^ and adjuvant treatment modality. We also used data on personal health habits, namely, alcohol consumption, smoking, and areca nut chewing, which were defined in our previous study^26^. The patients were subdivided into categories according to whether they kept none, one, or two or more of these habits^27^.

### Treatment protocol

All patients underwent curative surgery for OCSCC, and such surgery was accompanied by either bilateral or unilateral neck dissection. Patients with surgical defects were subjected to immediate reconstruction procedures executed by plastic surgeons. On the basis of the treatment guidelines for OCSCC established by our institution^9^, adjuvant therapy was administered within 6 weeks after the surgery if deemed necessary. Specifically, if necessary, the adjuvant intensity-modulated radiation therapy (RT) was applied to high-risk areas daily (dose: 2 Gy) for 5 days a week (cumulative dose: 66 Gy). Furthermore, if necessary, the chemotherapy was applied through the intravenous delivery of cisplatin-based regimen either weekly (dose: 40 mg/m^2^) or a triweekly (dose: 100 mg/m^2^), depending on the patient’s overall health and the oncologist’s assessment.

### Definition of mGNRI

We collected laboratory test results and weight and height data within 1 week before surgery to calculate the patients’ mGNRI values. In our central laboratory, the patients’ preoperative serum CRP levels (reference value: < 0.3 mg/dL) were measured using a Cobas 8000 instrument developed by Roche Hitachi (Rotkreuz, Switzerland), and their peripheral blood cell counts were measured through a Sysmex SE-9000 instrument developed by Sysmex (Kobe, Japan). The mGNRI was calculated as follows^23^:

$$ {\text{mGNRI }} = \, \left( {{14}.{89}/{\text{CRP }}\left[ {{\text{in mg}}/{\text{dL}}} \right]} \right) \, + { 41}.{7 } \times \, \left( {{\text{actual body weight}}/{\text{ideal body weight}}} \right). $$

In general, a CRP level of ≤ 0.3 mg/dL is deemed normal. Hence, in the calculations executed in our study, if a patient’s serum CRP level was < 0.3 mg/dL, we set it to 0.3 mg/dL^21^. Furthermore, we determined the ideal body weight in our calculations as that corresponding to an optimal BMI value of 22 kg/m^2^^23^.

### Follow-up and study endpoints

During the initial and second years following surgery, the patients underwent follow-up appointments every 2 to 3 months, and subsequent follow-ups were scheduled every 4 to 6 months. Our study’s primary outcomes comprised overall survival (OS; represented as the period spanning from the curative surgery date until either the occurrence of all-cause mortality or the last follow-up) and disease-free survival (DFS; represented as the period spanning from the curative surgery date until the first instance of cancer recurrence, distant metastasis, or last follow-up). We gathered survival data by conducting both medical chart reviews and telephone interviews. The period of follow-up assessments spanned from the curative surgery date to either December 31, 2019, or the date of death, whichever was earlier.

### Statistical analysis

Categorical variables are presented in terms of frequency and percentage. To ascertain whether our derived data were normally distributed, we employed the Kolmogorov–Smirnov test. For variables that we determined to be nonnormally distributed, we present their median and interquartile range (IQR). In addition, for continuous variables that we determined to be normally distributed, we present the mean and standard deviation. To explore the associations between mGNRI and clinicopathological characteristics, we used the chi-square test and Mann–Whitney *U* test to assess categorical and continuous variables, respectively. We analyzed receiver operating characteristic (ROC) curves and used Youden’s index to determine the optimal mGNRI threshold for predicting OS. The prognostic discriminations of mGNRI and its components, including BMI and serum CRP levels, were evaluated by calculating the area under the curve (AUC) for comparison. We used the Kaplan–Meier method to plot curves for OS and DFS, and we used the log-rank test to evaluate differences in survival between the curves. We used a stepwise Cox proportional hazards model to determine the prognostic factors that were independently associated with survival outcomes, and we derived the hazard ratio (HR) and 95% confidence interval (CI) for each factor. Variables that we determined to be significant in the univariable analysis (*p* < 0.1) were included in our multivariable analysis. All of the mentioned statistical analyses were executed in SPSS (version 23; IBM, Armonk, NY, USA), and a two-sided *p* value of < 0.05 indicated statistical significance.

We used the *rms* package (version 5.1-0; Vanderbilt University, Nashville, TN, USA) in R software to create a nomogram that was based on the significant prognostic factors noted in the multivariable analysis, and the endpoints were 3- and 5-year OS^28^. We ascertained our nomogram’s performance in OS prediction by comparing its predictive performance with that of the TNM staging system (when used alone) in terms of the concordance index (C-index). Furthermore, calibration plots were generated to assess the level of agreement between nomogram-predicted and actual survival outcomes.

## Results

### Baseline features

The 333 patients’ clinicopathological features and their associations with the mGNRI are described in Table 1. Men were noted to constitute a predominant proportion of the included patients (n = 300, 91.1%). Of the patients, 118 (35.4%) were ≥ 65 years old. The median (IQR) age and follow-up duration were determined to be 60 (52–69) years and 42.1 (21.0–67.8) months, respectively. The buccal mucosa (n = 107, 32.1%) and tongue (n = 130, 39.0%) were the two most prevalent tumor subsites. Furthermore, 74 (22.2%), 44 (13.2%), 50 (15.0%), and 165 (49.5%) patients had stage I, II, III, and IV OCSCC, respectively. Adjuvant therapy was administered to almost half of the patients; specifically, 124 (37.2%) patients received CRT, and 45 (13.5%) patients had adjuvant radiotherapy (RT). The majority of the patients (n = 272, 81.7%) had at least two of the outlined personal habits. Before undergoing surgery, 6 out of 333 patients (1.8%) required the nasogastric feeding tube due to significant malnutrition and compromised oral intake.

**Table 1:** Associations between modified geriatric nutritional risk index and clinicopathological features.

Variable	Total, *n* (%)	mGNRI < 73.3 (*n* = 146)	mGNRI ≥ 73.3 (n = 187)	*p*
Age (years)				0.451^a^
< 65	215 (64.6%)	91 (62.3%)	124 (66.3%)	
≥ 65	118 (35.4%)	55 (37.7%)	63 (33.7%)	
Sex				0.587^a^
Men	300 (90.1%)	133 (91.1%)	167 (89.3%)	
Women	33 (9.9%)	13 (8.9%)	20 (10.7%)	
Tumor subsite				0.542^a^
Tongue	130 (39.1%)	52 (35.6%)	78 (41.7%)	
Buccal mucosa	107 (32.1%)	46 (31.5%)	61 (32.6%)	
Others	96 (28.8%)	48 (32.9%)	48 (25.7%)	
Personal habit*				0.856^a^
No exposure	40 (12.0%)	18 (12.3%)	22 (11.8%)	
One exposure	21 (6.3%)	8 (5.5%)	13 (7.0%)	
Two or all exposure	272 (81.7%)	120 (82.2%)	152 (81.3%)	
Cancer stage				< 0.001^a^
I–II	118 (35.4%)	31 (21.2%)	87 (46.5%)	
III–IV	215 (64.6%)	115 (78.8%)	100 (53.5%)	
T status				< 0.001^a^
T1–T2	154 (46.2%)	44 (30.1%)	110 (58.8%)	
T3–T4	179 (53.8%)	102 (69.9%)	77 (41.2%)	
N status				< 0.001^a^
N0	216 (64.9%)	76 (52.1%)	140 (74.9%)	
N1–3	117 (35.1%)	70 (47.9%)	47 (25.1%)	
Presence of PNI				0.158^a^
No	252 (75.7%)	105 (71.9%)	147 (78.6%)	
Yes	81 (24.3%)	41 (28.1%)	40 (21.4%)	
Presence of ENE				< 0.001^a^
No	266 (79.9%)	100 (68.5%)	166 (88.8%)	
Yes	67 (20.1%)	46 (31.5%)	21 (11.2%)	
Presence of LVI				0.136^a^
No	311 (93.4%)	133 (91.1%)	178 (95.2%)	
Yes	22 (6.6%)	13 (8.9%)	9 (4.8%)	
Tumor grading				0.092^a^
W-D/M-D	294 (88.3%)	124 (84.9%)	170 (90.9%)	
P-D	39 (11.7%)	22 (15.1%)	17 (9.1%)	
Closest resection margin				0.046^a^
≥ 5 mm	244 (73.3%)	99 (67.8%)	145 (77.5%)	
< 5 mm	89 (26.7%)	47 (32.2%)	42 (22.5%)	
DOI ≥ 10 mm				< 0.001^a^
No	180 (54.1%)	57 (39.0%)	123 (65.8%)	
Yes	153 (45.9%)	89 (61.0%)	64 (34.2%)	
Treatment modality				< 0.001^a^
Surgery only	164 (49.2%)	53 (36.3%)	111 (59.4%)	
Surgery then RT	45 (13.5%)	20 (13.7%)	25 (13.4%)	
Surgery then CRT	124 (37.2%)	73 (50.0%)	51 (27.3%)	
CCI				0.354^a^
0	180 (54.1%)	73 (50.0%)	107 (57.2%)	
1	98 (29.4%)	45 (30.8%)	53 (28.3%)	
≥ 2	55 (16.5%)	28 (19.2%)	27 (14.4%)	
Albumin (g/dL), median (IQR)	4.4 (4.2–4.6)	4.3 (3.8–4.6)	4.5 (4.3–4.7)	< 0.001^b^
BMI (kg/m^2^), median (IQR)	25.0 (21.6–27.3)	23.8 (20.5–27.4)	25.3 (22.5–27.3)	0.077^b^
CRP (mg/dL), median (IQR)	0.35 (0.13–1.38)	1.88 (0.86–4.90)	0.15 (0.08–0.26)	< 0.001^b^
mGNRI, median (IQR)	82.6 (50.9–154.5)	48.6 (43.8–58.3)	141.6 (99.2–229.6)	< 0.001^b^

*CCI* Charlson comorbidity index, *CRT* chemoradiotherapy, *DOI* depth of invasion, *ENE* extranodal extension, *IQR* interquartile range, *LVI* lymphovascular invasion, *M-D* moderate differentiation, *mGNRI* modified geriatric nutritional risk index, *P-D* poor differentiation, *PNI* perineural invasion, *RT* radiotherapy, *W-D* well differentiation.

^a^Chi-square test.

^b^Mann–Whitney U test.

*Personal habits include cigarette smoking, alcohol consumption, and betel nut chewing.

### Associations between clinicopathological features and mGNRI

**Figure 1:**
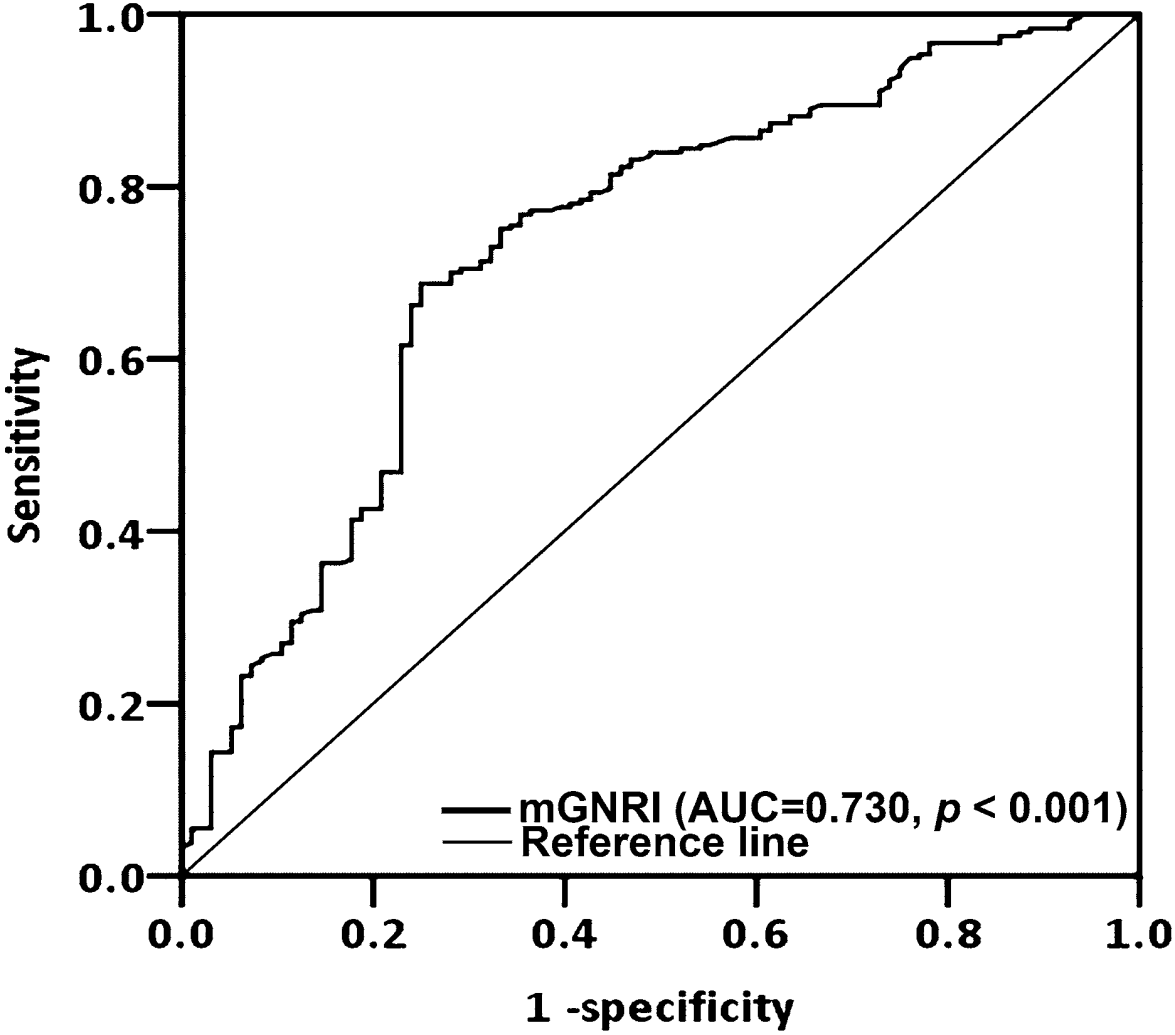
Cutoff value of mGNRI derived from receiver operating characteristic curve analysis. *AUC* area under the curve, *mGNRI* modified geriatric nutritional risk index.

Through ROC curve analysis, we determined the optimal mGNRI cutoff to be 73.3 (AUC 0.730; specificity, 75.0%; sensitivity, 68.8%; *p* < 0.001; Fig. 1). The AUC for preoperative mGNRI exceeded those for BMI (AUC, 0.611) and CRP (AUC, 0.712), suggesting that mGNRI possesses superior prognostic discrimination compared to BMI and CRP. Accordingly, using the cutoff of mGNRI, we subsequently divided the patients into a low-mGNRI group (< 73.3), which comprised 146 (43.8%) patients, and a high-mGNRI (≥ 73.3) group, which comprised 187 (56.2%) patients. The clinicopathological features of these groups are described in Table 1. We noted an mGNRI of < 73.3 to exhibit a significant association with the presence of ENE (*p* < 0.001), closest resection margin < 5 mm (*p* = 0.046), stage III–IV disease (*p* < 0.001), a need for adjuvant therapy (*p* < 0.001), DOI ≥ 10 mm (*p* < 0.001), a shorter median survival time (*p* < 0.001), and advanced T and N status (both *p* < 0.001). However, the mGNRI was not significantly associated with age, sex, tumor subsites, personal habits, PNI status, LVI status, tumor grading, or CCI.

### Association between mGNRI and OS

**Figure 2:**
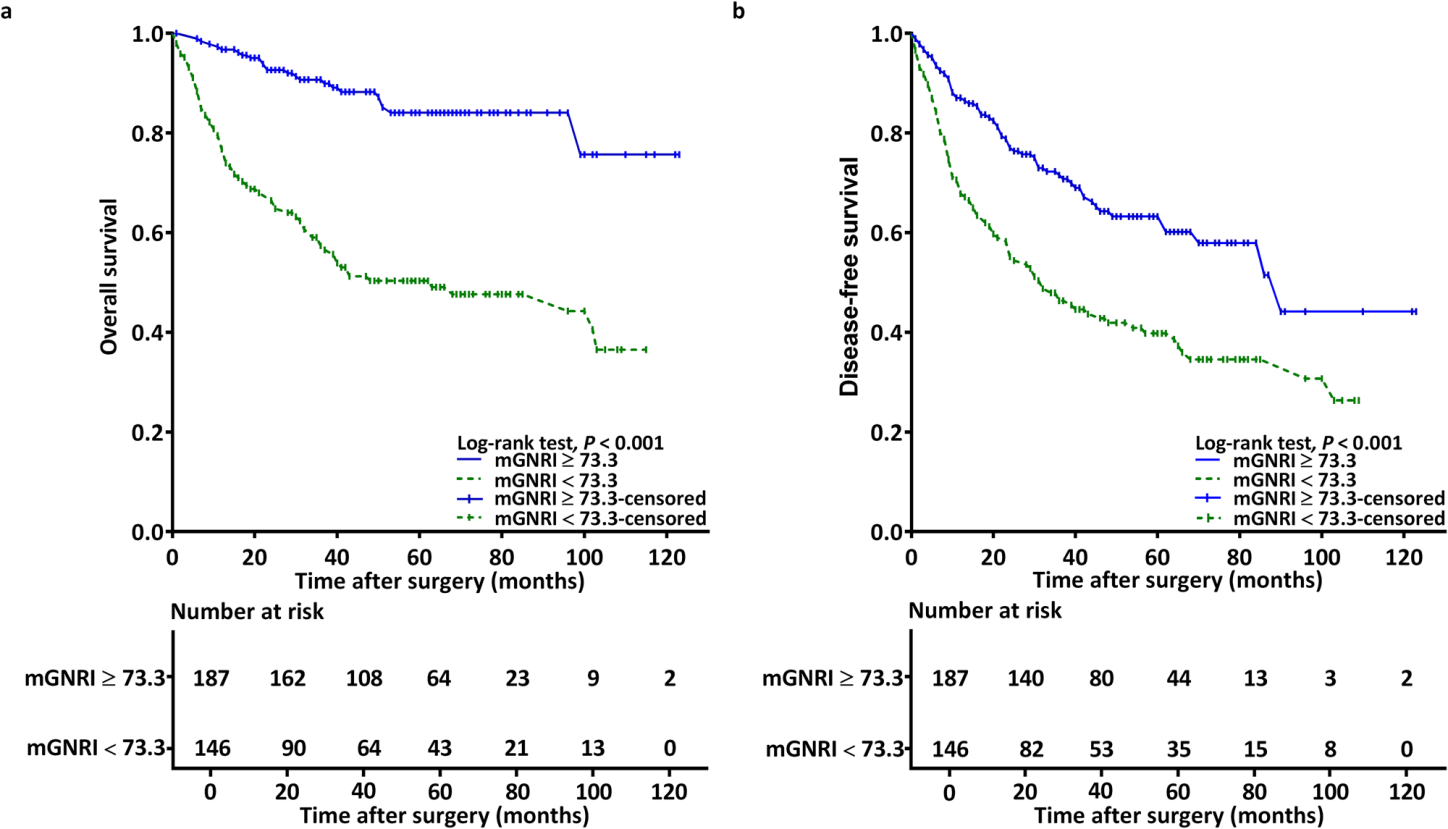
Kaplan–Meier curves for (**a**) overall survival and (**b**) disease-free survival stratified by preoperative mGNRI. An mGNRI value of < 73.3 was significantly associated with unfavorable outcomes. *mGNRI* modified geriatric nutritional risk index.

**Figure 3:**
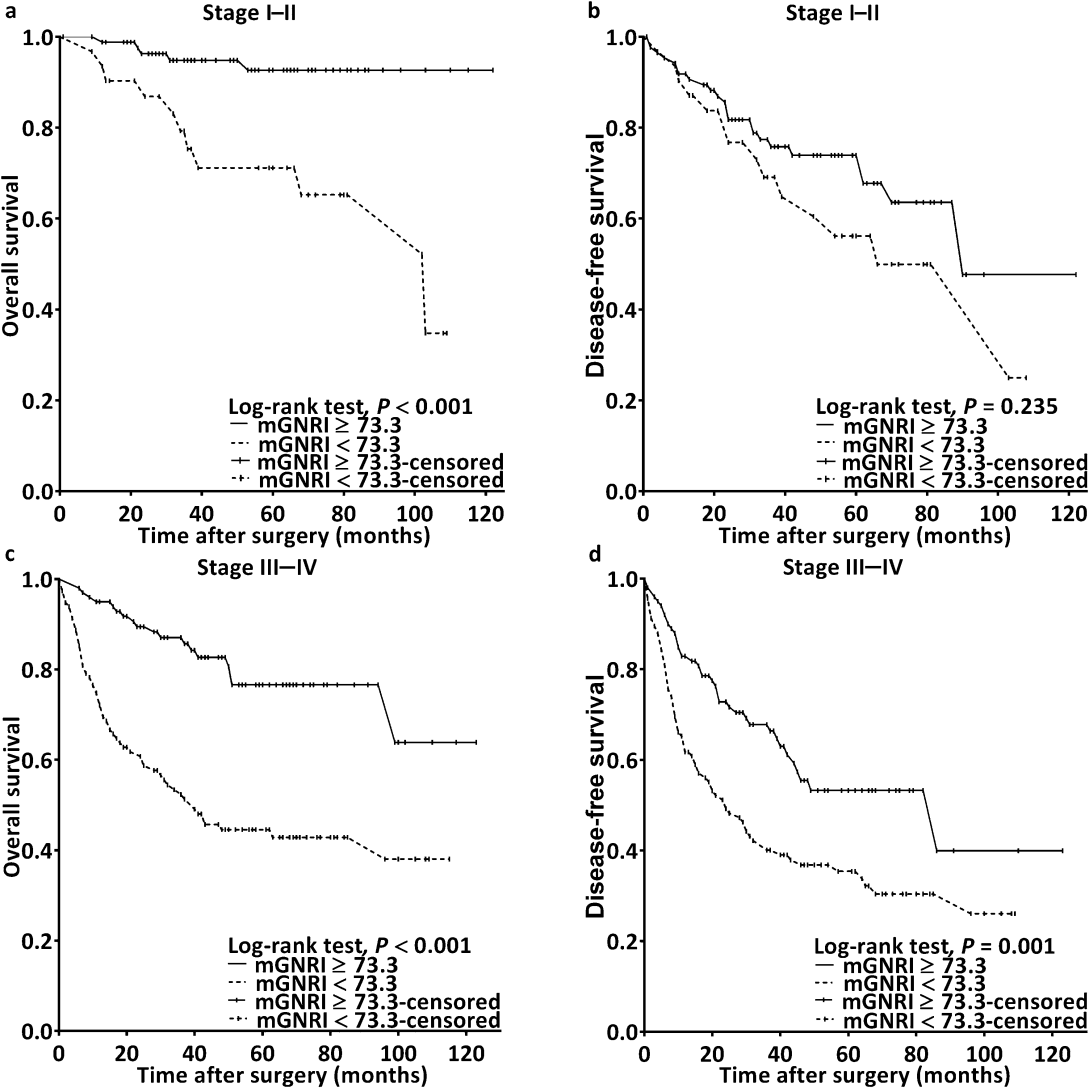
Kaplan–Meier survival curves for samples stratified by the mGNRI and cancer stage revealed a correlation between poorer overall survival and an mGNRI of < 73.3 in patients with (**a**) stage I–II and (**c**) stage III–IV (both *p* < 0.001) disease. Similar results were observed for disease-free survival in patients with (**d**) stage III–IV disease (p = 0.001), but not in those with (**b**) stage I–II disease (*p* = 0.235). *mGNRI* modified geriatric nutritional risk index.

During follow-up, 96 (28.8%) patients passed away. By assessing the plotted Kaplan–Meier curves, we noted the low- and high-mGNRI groups to differ significantly with respect to 5-year OS rates (50.7% vs. 87.2%; *p* < 0.001; Fig. 2a). Furthermore, the sample was stratified by cancer stage, and the analysis results revealed that a low mGNRI was consistently associated with worse OS in both early-stage (Fig. 3a) and advanced-stage (Fig. 3c) cancer (both *p* < 0.001). In our univariable analysis, we noted significant associations of unfavorable OS with mGNRI < 73.3, advanced-stage disease, presence of P-D, presence of LVI and PNI, CCI ≥ 1, and a need for adjuvant therapy (Table 2). In our multivariable analysis, we noted mGNRI < 73.3 to be an independent prognostic factor of unfavorable OS (HR 3.893; 95% CI 2.424–6.250; *p* < 0.001). Other independent factors included stage III–IV disease (HR 2.052; 95% CI 1.159–3.635; *p* = 0.014), the presence of PNI (HR 1.630; 95% CI 1.026–2.591; *p* = 0.039), P-D (HR 2.453; 95% CI 1.476–4.077; *p* < 0.001), and LVI (HR 2.443; 95% CI 1.307–4.566; *p* = 0.005).

**Table 2:** Univariable and multivariable analyses of clinicopathological variables for overall survival.

Variables		Univariable analysis		Multivariable analysis	
		HR (95% CI)	*p*	HR (95% CI)	*p*
Sex	Men vs. women	1.640 (0.758–3.459)	0.209		
Age (years)	≥ 65 vs. < 65	0.836 (0.545–1.283)	0.412		
Cancer stage	III–IV vs. I–II	3.320 (1.940–5.681)	< 0.001	2.052 (1.159–3.635)	0.014
PNI	Yes vs. no	2.368 (1.564–3.585)	< 0.001	1.630 (1.026–2.591)	0.039
LVI	Yes vs. no	3.883 (2.146–7.024)	< 0.001	2.443 (1.307–4.566)	0.005
Tumor grading	P-D vs. W-D/M-D	2.928 (1.797–4.770)	< 0.001	2.453 (1.476–4.077)	< 0.001
Closest margin (mm)	< 5 vs. ≥ 5	1.403 (0.917–2.147)	0.119		
Adjuvant therapy	Yes vs. no	2.931 (1.953–4.399)	< 0.001		
CCI	≥ 1 vs. 0	1.557 (1.040–2.330)	0.032	1.494 (0.991–2.252)	0.055
mGNRI	< 73.3 vs. ≥ 73.3	4.638 (2.918–7.370)	< 0.001	3.893 (2.424–6.250)	< 0.001

Variance Inflation Factor: Cancer stage: 1.119; PNI: 1.154; LVI: 1.045; Tumor grading: 1.018; CCI: 1.020; mGNRI: 1.040.

*CCI* Charlson comorbidity index, *CI* confidence interval, *HR* Hazard ratio, *LVI* lymphovascular invasion, *M-D* moderate differentiation, *mGNRI* modified geriatric nutritional risk index, *P-D* poor differentiation, *PNI* perineural invasion, *vs.* versus, *W-D* well differentiation.

### Association between mGNRI and DFS

By assessing our plotted Kaplan–Meier curves, we noted the low- and high-mGNRI groups to differ significantly with respect to 5-year DFS (39.7% vs. 66.3%; *p* < 0.001; Fig. 2B). Furthermore, the sample was stratified by cancer stage, and the results indicated a low mGNRI to be significantly associated with worse DFS in advanced-stage (*p* = 0.001; Fig. 3D) but not early-stage (*p* = 0.235; Fig. 3B) cancer. In our executed univariable analysis, we noted significant associations of poor DFS with LVI status, tumor grading, the need for adjuvant therapy, stage III–IV disease, and mGNRI < 73.3 (Table 3). In our multivariable analysis, the mGNRI < 73.3 (HR 1.803, 95% CI 1.292–2.517, *p* < 0.001), P-D (HR 2.011, 95% CI 1.302–3.108,* p* = 0.002), and late-stage disease (HR 1.734, 95% CI 1.186–2.535, *p* = 0.005) were noted to be independent predictors of unfavorable DFS.

**Table 3:** Univariable and multivariable analyses of clinicopathological variables for disease-free survival.

Variables		Univariable analysis		Multivariable analysis	
		HR (95% CI)	*p*	HR (95% CI)	*p*
Sex	Men vs. women	1.605 (0.884–2.915)	0.120		
Age (years)	≥ 65 vs. < 65	0.719 (0.508–1.017)	0.062		
Cancer stage	III–IV vs. I–II	2.049 (1.417–2.961)	< 0.001	10.734 (1.186–2.535)	0.005
PNI	Yes vs. no	1.387 (0.969–1.987)	0.074		
LVI	Yes vs. no	2.050 (1.157–3.635)	0.014	10.754 (0.984–3.125)	0.057
Tumor grading	P-D vs. W-D/M-D	2.056 (1.335–3.167)	0.001	20.011 (1.302–3.108)	0.002
Closest margin (mm)	< 5 vs. ≥ 5	1.284 (0.911–1.810)	0.153		
Adjuvant therapy	Yes vs. no	1.759 (1.276–2.424)	0.001		
CCI	≥ 1 vs. 0	1.021 (0.741–1.408)	0.898		
mGNRI	< 73.3 vs. ≥ 73.3	2.055 (1.485–2.843)	< 0.001	10.803 (1.292–2.517)	< 0.001

Variance Inflation Factor: Cancer stage: 1.054; LVI: 1.012; Tumor grading: 1.002; mGNRI: 1.043.

*CCI* Charlson comorbidity index, *CI* confidence interval, *HR* hazard ratio, *LVI* lymphovascular invasion, *M-D* moderate differentiation, *mGNRI* modified geriatric nutritional risk index, *P-D* poor differentiation, *PNI* perineural invasion, *vs.* versus, *W-D* well differentiation.

### Stratified analysis of OS prediction

**Figure 4:**
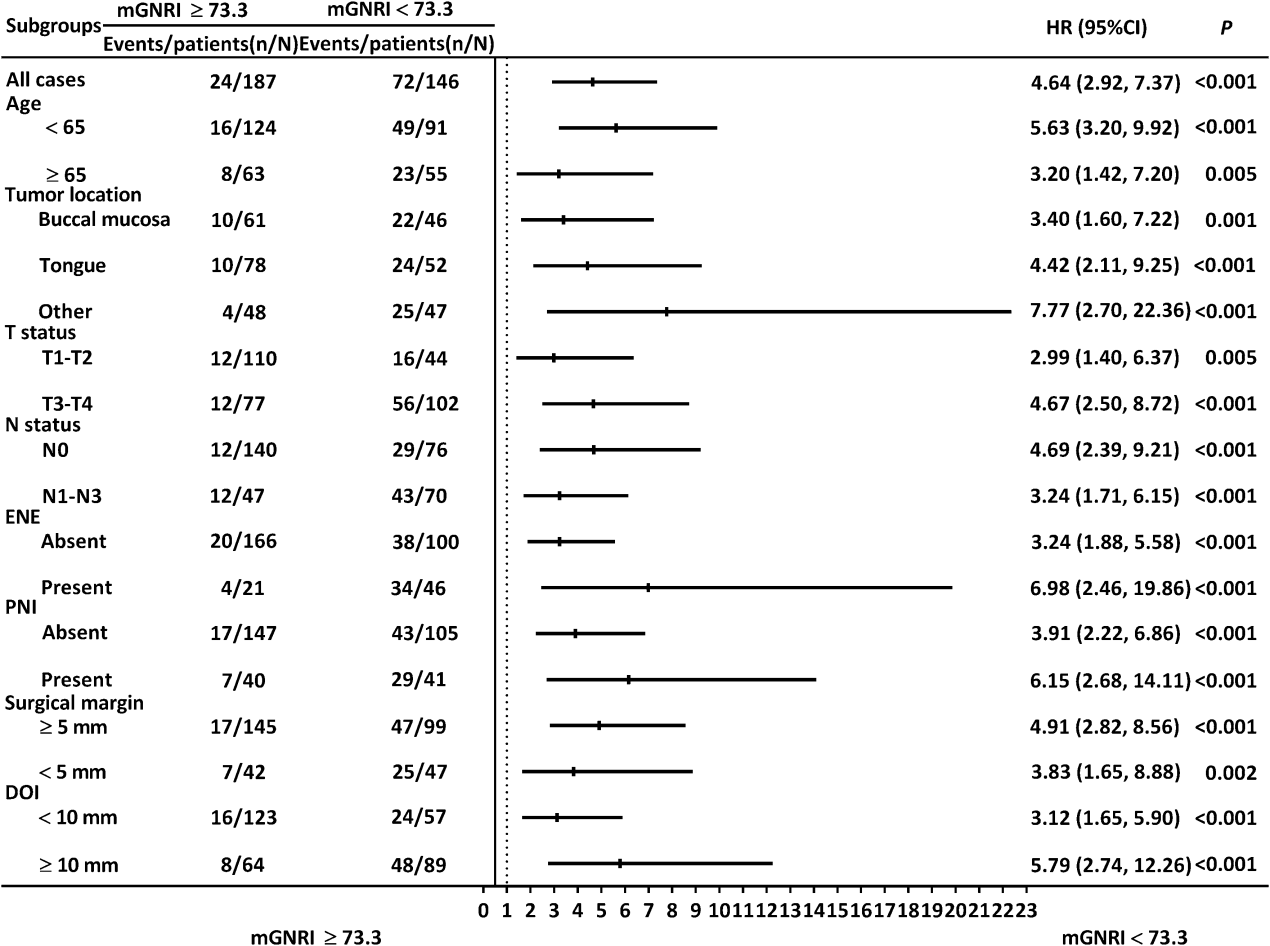
Stratified analysis examining the associations between the mGNRI and OS. A consistent trend was observed across all subgroups. *CI* confidence interval, *DOI* depth of invasion, *ENE* extranodal extension, *HR* Hazard ratio, *mGNRI* modified geriatric nutritional risk index, *PNI* perineural invasion.

The associations between the mGNRI and OS remained consistent and significant when the patients were segmented by tumor subsite, age (≥ 65 or < 65 years), N status (N0 or N1–N3), T status (T1–T2 or T3–T4), ENE status (absent or present), PNI status (absent or present), closest surgical margin (≥ 5 mm or < 5 mm), and DOI status (≥ 10 mm or < 10 mm; Fig. 4).

### Nomogram for OS prediction

**Figure 5:**
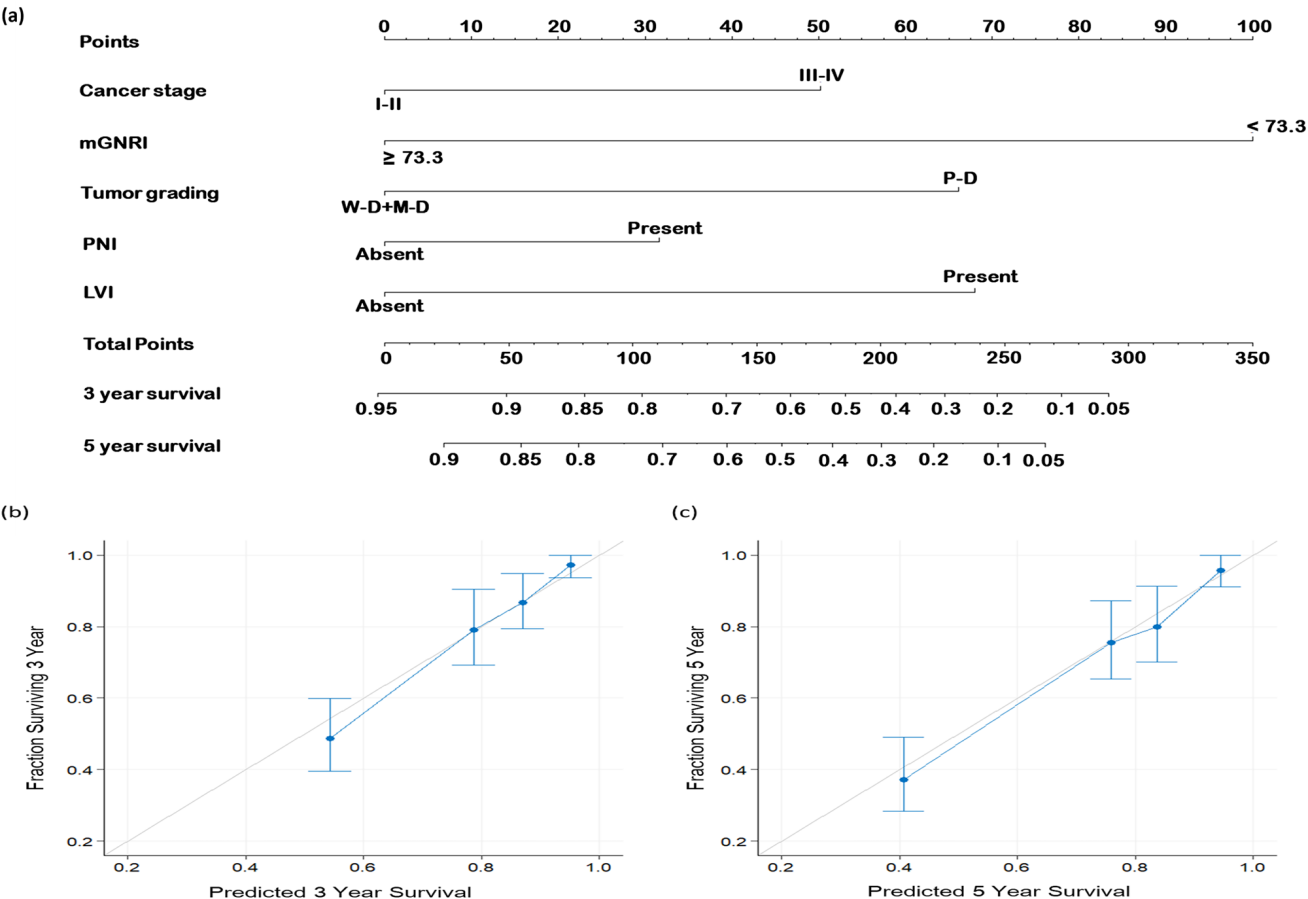
Predictive nomogram. (**a**) Nomogram designed for predicting OS based on the mGNRI and the independent prognostic factors identified through multivariable analysis. The contribution of each variable’s risk level is indicated by the line segment and its corresponding points. The total points were calculated by summing the points for each variable. To determine the likelihood of 3-year and 5-year OS, a vertical line was drawn from the calculated total points. Calibration plots for (**b**) 3-year and (**c**) 5-year OS are displayed. The light gray line at a 45° angle represents perfect predictive accuracy, and the blue line represents the predictive outcomes of the nomogram. The nomogram’s performance, along with the 95% confidence intervals for OS predictions, is presented as blue dots with bars. *LVI* lymphovascular invasion, *M-D* moderate differentiation, *mGNRI* modified geriatric nutritional risk index, *P-D* poor differentiation, *PNI* perineural invasion, *W-D* well differentiation.

Our constructed nomogram included the significant prognostic factors, namely, mGNRI, cancer stage, tumor grading, PNI, and LVI, derived from our multivariable analysis (Fig. 5a). The C-index value derived for our mGNRI-based nomogram were 0.781 (95% CI 0.751–0.813), whereas the C-index value derived for the AJCC stage-based nomogram was 0.631 (95% CI 0.605–0.658). To further verify our nomogram’s accuracy, we drew calibration plots illustrating the probabilities of 3- and 5-year OS (Fig. 5b,c, respectively). Both calibration plots were noted to closely follow a 45° diagonal line, signifying the high calibration accuracy of the constructed nomogram.

## Discussion

We found the mGNRI to be a potential biomarker in patients receiving surgery for OCSCC. According to our Kaplan–Meier analyses, preoperative mGNRI < 73.3 was determined to exhibit a marked association with a shorter 5-year OS and DFS. The results of Cox analyses revealed that a low mGNRI (< 73.3) serves as an independent risk factor for both OS and DFS, increasing the associated risks by 3.893-fold and 1.803-fold, respectively. In our subgroup analyses, the prognostic value of the mGNRI in terms of OS remained unchanged in various subgroups, indicating its robustness. The significant associations of a low mGNRI with several adverse clinicopathological features in our analyses underscore the importance of assessing the preoperative nutritional and inflammatory statuses of patients when determining OCSCC aggressiveness. Possible explanations for these associations include the following: (1) Extensive tumor volume and metastatic nodal involvement could potentially induce heightened cancer-related systemic inflammation, leading to elevated CRP levels, consequently resulting in a low mGNRI. (2) Increased tumor burden and potential cachexia may induce involuntary loss of adipose tissue and skeletal muscle mass, leading to decreased body weight and a lower mGNRI^29^. We also conducted several analyses to validate the independent prognostic significance of mGNRI in managing OCSCC. Initially, we assessed mGNRI’s ability to predict OS using Kaplan–Meier estimates stratified by cancer stage. Moreover, we identified significant and consistent associations between mGNRI and OS in subgroup analysis. Lastly, employing stepwise multivariable analysis assisted in mitigating multicollinearity, which arises from high correlations among predictor variables. Our results support our hypothesis and demonstrate that preoperative mGNRI is a promising biomarker for predicting long-term prognosis in OCSCC. We believe that the mGNRI should be included in future oncology research and clinical practice because data for calculating the mGNRI are inexpensive and cost-effective. Future research should investigate whether the prognostic performance of the mGNRI holds for other treatment modalities and whether changes in the mGNRI over time are indicative of the efficacy of anti-cancer therapies.

Recent research has investigated the applicability of the mGNRI for the prediction of prognosis in cancer. Data from 128 patients with esophageal cancer demonstrated that a low mGNRI of < 70 was an independent negative prognostic factor for OS and relapse-free survival^21^. Teruhisa et al. explored the association between pretreatment mGNRI and prognosis among 137 patients with pancreatic cancer and suggested that the mGNRI is a superior prognostic indicator compared with the GNRI^23^. In a prospective cohort study involving 3833 patients who underwent chemotherapy for various types of cancer, the mGNRI outperformed the GNRI as a prognostic indicator^22^. The superior prognostic performance of the mGNRI relative to the GNRI may be because the mGNRI, as a modified version of the GNRI that accounts for not only nutritional status but also systemic inflammatory responses, is a more comprehensive indicator of a patient’s overall condition. Our study demonstrated that the mGNRI is a robust predictor of prognosis in OCSCC, affirming its wide applicability across different cancer types. Nevertheless, the mGNRI is not a cancer-specific biomarker, and its clinical utility for cancer prognosis requires further investigation through large-scale prospective studies.

The mechanisms underlying the correlation between a low mGNRI and poor survival outcomes remain unclear. Generally, adjuvant therapy improves disease control and survival outcomes in OCSCC patients with unfavorable pathologic primary and nodal characteristics. In this study, patients with lower mGNRI scores displayed more adverse clinicopathological features, indicating a greater requirement for adjuvant treatment in this group. This observation may partially explain the association between mGNRI and survival outcomes. Furthermore, the mGNRI is based on levels of serum CRP, an acute-phase protein that arises in response to interleukin (IL)-6 secretion by T cells and macrophages, serving as a reflection of systemic inflammatory responses^30^. IL-6 is frequently upregulated in different cancer types, including OCSCC^31^, and heightened IL-6 levels can potentially stimulate nodal and distant metastasis through PI3K/AKT/mTOR pathway activation^32^. Regarding the tumor microenvironment, insufficient blood supply to the central part of tumor may lead to tumor necrosis and inflammation as the tumor grows^5^. In addition, the direct extension and infiltration of cancer cells can engender tissue inflammation and damage^33^. Hence, the increase in CRP levels following IL-6 upregulation may not only represent a reaction to tumor progression but also signify tissue inflammation and damage, which could potentially function as a mechanism linking low mGNRI levels and unfavorable survival outcomes. Additionally, the association between malnutrition, which is often reflected by decreased BMI, and cancer prognosis has been thoroughly examined across various cancer types^34^. Patients with OCSCC may exhibit lower BMI values than do individuals in the general population owing to the effects of the cancer and cancer-associated dysphagia; therefore, the mGNRI provides additional insight into patients’ condition by accounting for the patients’ ideal weight, thereby offering a more objective evaluation of the patients’ weight. A previous study observed that being underweight at head and neck cancer (HNC) diagnosis was an independent risk factor for unfavorable prognosis^35^; conversely, overweight was associated with the likelihood of complete response after CRT^36^. These observations could be linked to cancer-related chronic wasting, oxidative stress, and the high protein metabolism induced by cancer cachexia^37^. The aforementioned study results hint at the probable mechanisms through which a lower mGNRI is associated with poorer prognosis in OCSCC; however, further study is required to elucidate this mechanism.

The AJCC staging system, an extensively employed system for cancer staging and survival prediction, predominantly concentrates on tumor characteristics; nevertheless, patients with the same disease stage frequently differ in their prognoses. A growing body of evidence suggests that patient-related factors, such as nutritional status and systemic inflammatory responses, are significantly associated with survival outcomes in OCSCC^26,38,39^, and must thus not be neglected in predictions of survival in OCSCC. Recognizing the potential of the mGNRI as a valuable indicator for assessing both host nutrition status and inflammatory responses, we have created an mGNRI-based nomogram that successfully predicts individualized OS. We also drew calibration plots to visually demonstrate the nomogram’s accuracy; the plots revealed a close alignment between the nomogram-predicted OS probabilities and actual survival outcomes. These findings confirm the excellent predictive performance of the mGNRI-based nomogram and support the clinical utility of preoperative mGNRI in the early stages of OCSCC treatment.

The mGNRI aids cancer management with potential therapeutic implications. Patients with lower preoperative mGNRI levels may exhibit increased systemic inflammation and nutritional risk; this is a multifaceted condition that necessitates holistic treatment strategies^40^. The favorable effects of nutritional assistance for patients with HNC have been consistently reported in studies on the topic. For example, in a double-blind, randomized trial involving 110 HNC patients who underwent CRT, the provision of immunonutrition enhanced survival outcomes^41^. This effect was particularly significant in patients with nasopharyngeal carcinoma. The possible explanation for this observation is that the immunonutrition may mitigate the risk of severe mucositis, support overall nutritional status during CRT, and consequently improve treatment compliance and response^42^. An increase in tumor-related inflammation, as indicated by a low mGNRI levels, may provide valuable insights into potential therapies. A study indicated that nonsteroidal anti-inflammatory drugs (NSAIDs) have the potential to act as chemopreventive agents for OCSCC by modifying the expression of IL-6 and the gene expression of chemokines^43^. Moreover, a comprehensive meta-analysis involving 17 studies investigated the effects of NSAIDs on the survival outcomes of HNC^44^. The results demonstrated the protective role of NSAIDs in lowering the risk of HNC and the ability of NSAIDs to improve cancer-specific survival and ameliorate disease recurrence. These findings suggest that patients with low mGNRI levels may respond favorably to nutritional interventions, anti-inflammatory agents, and personalized treatment strategies. This suggestion should be investigated in future studies.

This study has the following strengths. First, our executed study is the first to probe the application of the mGNRI for prognosis in OCSCC. Second, the study included a relatively large number of patients with OCSCC who received the same treatment protocol. Third, the study included a range of clinicopathological variables to mitigate confounding. Finally, this study constructed a nomogram to demonstrate the clinical usefulness of the mGNRI in predicting individualized OS. This study has some limitations. First, the single-institutional and retrospective design may introduce bias and limit the generalizability of our findings. Second, we did not validate our findings with an independent dataset. In addition, the male-to-female ratio in this study was 9:1, which aligns with previous findings demonstrating that OCSCC primarily impacts males worldwide^45^. This trend could be attributed to the higher prevalence of risky behaviors, such as smoking and areca nut chewing, among males compared to females in Taiwan^46^. Given the potential differences in CRP and BMI cutoff values between genders, future studies could establish sex-specific thresholds for mGNRI to enhance its accuracy in managing OCSCC. Given these limitations, large-scale and prospective studies are required to corroborate our findings.

In conclusion, the preoperative mGNRI, as an indicator of cancer-associated malnutrition and inflammation, exhibits potential as a prognostic biomarker for patients undergoing surgical treatment for OCSCC. The study also constructed a nomogram based on the mGNRI and significant clinicopathological factors; it provides precise predictions of OS, enhancing the clinical applicability of the mGNRI and facilitating the delivery of early and personalized treatment. Furthermore, the mGNRI is inexpensive and easy to measure and can be applied by both researchers and clinicians working with cancer.

## Data Availability

The datasets used and analyzed during the current study are available from the corresponding author upon reasonable request.
